# Roles Played by the Na^+^/Ca^2+^ Exchanger and Hypothermia in the Prevention of Ischemia-Induced Carrier-Mediated Efflux of Catecholamines into the Extracellular Space: Implications for Stroke Therapy

**DOI:** 10.1007/s11064-019-02842-0

**Published:** 2019-07-25

**Authors:** M. Lakatos, M. Baranyi, L. Erőss, S. Nardai, T. L. Török, B. Sperlágh, E. S. Vizi

**Affiliations:** 1 Institute of Experimental Medicine, Hungarian Academy of Sciences, Budapest, Hungary; 2 Department of Pharmacology and Pharmacotherapy, Semmelweis University, Budapest, Hungary; 3 National Institute of Clinical Neuroscience, Budapest, Hungary

**Keywords:** Ischemia, Dopamine release, Dopamine, Non-vesicular, DOPAL, Toxic, Na^+^/Ca^2+^ exchanger, Monoamine transporter, Extracellular space, Hypothermia

## Abstract

The release of [^3^H]dopamine ([^3^H]DA) and [^3^H]noradrenaline ([^3^H]NA) in acutely perfused rat striatal and cortical slice preparations was measured at 37 °C and 17 °C under ischemic conditions. The ischemia was simulated by the removal of oxygen and glucose from the Krebs solution. At 37 °C, resting release rates in response to ischemia were increased; in contrast, at 17 °C, resting release rates were significantly reduced, or resting release was completely prevented. The removal of extracellular Ca^2+^ further increased the release rates of [^3^H]DA and [^3^H]NA induced by ischemic conditions. This finding indicated that the Na^+^/Ca^2+^ exchanger (NCX), working in reverse in the absence of extracellular Ca^2+^, fails to trigger the influx of Ca^2+^ in exchange for Na^+^ and fails to counteract ischemia by further increasing the intracellular Na^+^ concentration ([Na^+^]_i_). KB-R7943, an inhibitor of NCX, significantly reduced the cytoplasmic resting release rate of catecholamines under ischemic conditions and under conditions where Ca^2+^ was removed. Hypothermia inhibited the excessive release of [^3^H]DA in response to ischemia, even in the absence of Ca^2+^. These findings further indicate that the NCX plays an important role in maintaining a high [Na^+^]_i_, a condition that may lead to the reversal of monoamine transporter functions; this effect consequently leads to the excessive cytoplasmic tonic release of monoamines and the reversal of the NCX. Using HPLC combined with scintillation spectrometry, hypothermia, which enhances the stimulation-evoked release of DA, was found to inhibit the efflux of toxic DA metabolites, such as 3,4-dihydroxyphenylacetaldehyde (DOPAL). In slices prepared from human cortical brain tissue removed during elective neurosurgery, the uptake and release values for [^3^H]NA did not differ from those measured at 37 °C in slices that were previously maintained under hypoxic conditions at 8 °C for 20 h. This result indicates that hypothermia preserves the functions of the transport and release mechanisms, even under hypoxic conditions. Oxidative stress (H_2_O_2_), a mediator of ischemic brain injury enhanced the striatal resting release of [^3^H]DA and its toxic metabolites (DOPAL, quinone). The study supports our earlier findings that during ischemia transmitters are released from the cytoplasm. In addition, the major findings of this study that hypothermia of brain slice preparations prevents the extracellular calcium concentration ([Ca^2+^]_o_)-independent non-vesicular transmitter release induced by ischemic insults, inhibiting Na^+^/Cl^−^-dependent membrane transport of monoamines and their toxic metabolites into the extracellular space, where they can exert toxic effects.

## Introduction

Cerebral ischemia is a pathological condition, during which blood flow to neurons is insufficient or completely blocked. Cerebral ischemia can be focal, such as when a brain artery is occluded, or global, such as after cardiac arrest. Ischemic stroke is the leading cause of brain dysfunction. However, because the mechanism of stroke pathogenesis has not been clarified to date, no effective medical treatments for reversing sensory, motor and cognitive impairments due to stroke have been devised [1, 2]. Stroke is the second most prevalent cause of death and is the most common cause of permanent disability in adults [3].

The brain is a wired instrument, and its neurons process cabled information through synapses; however, neurons are also able to communicate with each other nonsynaptically, without synaptic contact [4–13]. Evidence has shown that nonsynaptic receptors and transporters are characterized by high affinity [5, 6] and have many implications for psychiatric issues, such as depression, changes in mood, changes in appetite, and psychiatric disorders. Therefore, it has been suggested that many drugs used to treat psychiatric diseases may exert their effects through diffusion into extracellular spaces, where they may mimic or influence the effects of endogenous ligands [5, 6]. Nonsynaptic chemical communication between neurons and between neurons and their target cells, via both pre- and postsynaptic sites, were also identified during the excellent studies by Agnati and Fuxe [14, 15], who used the term ‘volume transmission’ to denote non-synaptic transmission.

Extracellular Ca^2+^ concentration ([Ca^2+^]_o_)-dependent release represents vesicular exocytotic release that is evoked by axonal action potentials, whereas the [Ca^2+^]_o_-independent release that can be measured at rest has been identified as being a non-exocytotic release from the cytoplasm, caused by the reversal of transporter activity [16–18]. Ischemic conditions (oxygen and glucose deficiencies) have previously been shown to result in the excessive production of reactive oxygen species (ROS) [19–22], oxidative stress (primarily during the reperfusion period) and the release of transmitters from the cytoplasm in a [Ca^2+^]_o_-independent manner [23–26]. Oxidative stress has been implicated as being the mechanism through which ischemia results in brain injury [21, 22]. This type of transmitter release from nonsynaptic varicosities [5, 12] into the extracellular space [21, 22] occurs even when the classical Ca^2+^-dependent vesicular transmission is inhibited or impaired. Under this condition membrane transporters, when acting in reverse, are able to release signaling molecules from the cytoplasm, causing an increase in the extracellular concentrations of transmitters. This paper addresses this type of release and the possible methods for preventing it.

When a train of action potentials (APs) reaches a nerve terminal, any residual [Ca^2+^] above 500 nM begins to be extruded by the NCX, which has a low affinity (K_d_ = 500 nM) and a high capacity (5 × 10^3^ Ca^2+^/s) for Ca^2+^ [27]. However, when intracellular Na^+^ levels increase excessively or strong membrane depolarization occurs, the NCX reverses and exports three Na^+^ ions for each imported Ca^2+^ ion (the “reverse” mode of NCX activity [27, 28]). The expression patterns of 2 NCX isomers (NCX2 and NCX3) are primarily restricted to the brain, where they are presynaptically localized to synaptic terminals [29–31] and control the cytoplasmic Ca^2+^ levels in the nerve terminal, which increase from 0.1 (during resting conditions) to 20 µM [32, 33] (during an action potential).

Various studies have demonstrated that an NCX acting in reverse promotes the additional entry of Ca^2+^ [34] and apoptotic neuronal death during stroke and brain trauma. In response to neurotoxic glutamate release during ischemic insults [26, 35], when amino acid transporters (EAATs) are also inhibited, the excessive influx of Na^+^ and Ca^2+^ [36] results in the reverse activation of the NCX and excitotoxicity. These findings indicate that the NCX may be involved in neurotoxicity.

In addition, there is convincing evidence that cytosolic dopamine (DA) is highly toxic following deamination by MAO, which converts DA to 3,4-dihydroxyphenylacetaldehyde (DOPAL) [37] and hydrogen peroxide (H_2_O_2_) [20], both of which have been shown to be neurotoxic [38, 39]. Cytosolic DA can also be auto-oxidized to form ROS (hydroxyl radicals, superoxide and H_2_O_2_) [20, 40]. Therefore, in this study, we measured the levels of DA and noradrenaline (NA) uptake and release from striatal and cerebral cortical slices, both at rest and in response to axonal activity, under normal and ischemic conditions and in the presence and absence of Ca^2+^ ions in the perfusion solution. Using high-performance liquid chromatography (HPLC) combined with scintillation spectrometry, we also studied whether the toxic metabolites of DA were released. The role played by the plasmalemmal exchanger, which is crucial for the clearance of cytoplasmic Ca^2+^, during neurotoxicity and targeted treatments is controversial [28, 41]; therefore, we attempted to influence the excessive release of transmitters (DA and NA) by using NCX inhibitors under ischemic conditions, during which [Na^+^]_i_ is high, and the NCX is expected to operate in reverse. We also studied the effects of Ca^2+^ removal on the cytoplasmic release of transmitters in response to ischemia when extraneuronal Ca^2+^ would normally be exchanged for Na^+^ and predicted to decrease [Na^+^]_i_.

Recently, 2-[2-[4(-nitrobenzyl-oxy)phenyl]ethyl]isothiourea (KB-R7943) was reported to potently inhibit both forms of NCX without affecting the Na^+^-dependent transport systems [42]. Therefore, we studied the effects of KB-R7943 on the release of monoamines in response to ischemia.

Lowering the temperature depresses the metabolic rates [43] of neurons and prevents the resting release of transmitters induced by transporters operating in reverse [44–46]. Furthermore, in clinical practice, therapeutic hypothermia has been used with moderate levels of success, not only for stroke and myocardial infarction but also for neonatal encephalopathy [47, 48]. Mild to moderate hypothermia has been shown [49–51] to have neuroprotective effects against ischemic and hypoxic insults [52, 53] in adults, in newborns [51, 54–56] and in animal experiments [57–59]. Therefore, we also studied the effects of hypothermia, induced by cooling slice preparations, on transmitter (DA and NA) release evoked by ischemia from rat striatal and cortical slices.

For the first time, this paper provides neurochemical evidence regarding the mechanism through which hypothermia inhibits the resting release of catecholamines from the cytoplasm, which may prevent the toxic effects of monoamine metabolites (aldehydes) in the extracellular space. The finding that hypothermia preserves the machinery necessary for transmitter function, even under hypoxic conditions, in human cortical slices provides further evidence of its neuroprotective effects.

## Materials and Methods

### Preparation of Rat Striatal and Cortical Slices

All experiments using Wistar rats (37–48 days old) were conducted with the permission of the local Animal Care Committee. The rats were lightly anaesthetized with diethyl ether and decapitated. The brain was removed and immediately placed in ice-cold Krebs solution that was continuously mixed with 95% O_2_ and 5% CO_2_. The Krebs solution used in this study contained the following in mmol/l: NaCl, 113; KCl, 4.7; CaCl_2_ 2.5; KHPO_4_, 1.2; MgSO_4_, 1.2; NaHCO_3_, 25; glucose, 11.5; ascorbic acid, 0.3; and Na_2_EDTA, 0.03. The striatum or frontal cortex was dissected and cut transversely into 400-µm slices using a McIlwain tissue slicer. The slice preparations used in this study were previously described [60]. In our experiments, we did not compensate for the changes in osmolarity produced by the removal of glucose because we did not observe any differences in the release rates when glucose was not replaced [61].

In a series of experiments, olfactory bulb slice preparations were used, as described previously [62]. The olfactory bulb preparation is advantageous because the release of dopamine is dominant in this preparation.

### Release of [^3^H]dopamine/[^3^H]noradrenaline

Briefly, after the striatal and frontal cortical slices were placed in Krebs solution, the tissue slices were incubated in 1 ml Krebs solution in the presence of either 5 μCi/ml [^3^H]DA or 5 µCi/ml [^3^H]NA at 37 °C for 45 min. After the loading period, the tissue was washed five times with Krebs solution, transferred to a thermoregulated superfusion apparatus [63], and superfused at 37 °C with a Krebs solution saturated with 95% O_2_ + 5% CO_2_. After a 20 min pre-perfusion treatment (flow rate: 0.5 ml/min), the effluent samples were collected every three minutes. During the collection of the 3rd (9 min) and the 13th (39 min) samples, the tissue was stimulated (S_1_ and S_2_, respectively) with square-wave impulses, using a Grass S88 stimulator (Astro-Med, West Warwick, RI, USA). The striatal slices were stimulated at 30 V, 2 Hz, 2 ms for 2 min (240 shocks), and the cortical slices were stimulated at 20 V, 2 Hz, 2 ms for 3 min (360 shocks). These stimulation parameters proved to be supramaximal. Unless otherwise stated, the experimental conditions were not changed after the control collection periods, and the release of radioactivity was measured and expressed in disintegrations per gram of tissue (Bq/g). At the end of the experiment, the tritium contents of the tissues were determined, as previously described [64]. Using a computer programme, the fractional release (FR) values for [^3^H]DA (FRS_1_) and [^3^H]NA (FRS_2_) in response to stimulation were calculated as percentages of the total radioactivity present in the tissue at the beginning of stimulation; the basal resting release was determined during the collection periods before (FRR_1_) and during ischemia (FRR_2_). The fractional release (FR) values during the R_1_ resting (FRR_1_) or S_1_ stimulation (FRS_1_) periods served as internal controls for the calculations of the FRR_2_/FRR_1_ and FRS_2_/FRS_1_ ratios. Samples used to calculate R_1_ and R_2_ are indicated in legends. The extent of the cytoplasmic DA release during ischemia varied in experiments carried out at different time points, but always remained very high. Therefore, we tried to carry out experiments that addressed a problem within several weeks.

To prevent the metabolism of catecholamines by MAO during release experiments, the Krebs solution contained 10 µM pargyline. A thermoelectric device (Frigomix, B. Braun, Germany) was used to rapidly change the temperature of the bath solution. Ischemia was simulated by placing tissue slices in Krebs solution lacking glucose and mixing them with 95% N and 2.5% CO_2_ [65]; ischemic conditions were simulated starting with the collection of the 7th fraction and maintained until the end of the experiments. When Ca^2+^-free Krebs solution was used, CaCl_2_ was omitted, and 1 mM EGTA was added to the Krebs solution.

The released [^3^H]NA and [^3^H]DA levels were measured using a Packard-Canberra TR 1900 liquid scintillation counter. The advantage of our acute slice preparations being maintained in a microperfusion system (100 µl) is that the slices are very thin (400 µm), and exchanging the perfusion fluid within the extracellular space is easily performed; furthermore, drugs may easily exert their effects, and oxygen and glucose removal is more efficient.

### Chromatographic Analysis of Dopamine and Its Metabolites

The method described by Baranyi et al. [64] was used to study the metabolism of DA in slices loaded with 5 µC [^3^H]DA. Under this condition, the Krebs solution did not contain pargyline. The slices were superfused, and the collected 3 min samples were analysed by high-performance liquid chromatography (HPLC) combined with liquid scintillation spectrometry.

### Human Frontal Cortical Slice Preparation

Human frontal or temporalis cortical samples (n = 8) were obtained from bulk brain tissue that was removed during elective neurosurgery conducted on 8 patients. The tissues were randomly selected from brain surgeries that were performed at the National Institute of Clinical Neuroscience (Budapest, Hungary) and were used for histology. This study was not limited to any type of surgical procedure or day of the week, and no tissue was removed for the express purpose of this study. The ages of the patients varied from 31 to 68 years (mean 48.2 ± 10.8 years old). Daily medications were continued until surgical intervention. Standard premedication and induced anaesthesia procedures (patients were ventilated with a mixture of 40% oxygen plus 60% nitrous oxide) were used. Total intravenous anaesthesia, 1% propofol at a rate of 20 ml/h, was administered by perfusion. Fentanyl, at 0.05 mg for 20–30 min, and 2 mg cisatracurium every 1.5 h were also used. The patients underwent multimodal monitoring. The specimens were used for in vitro experiments within 2 h of collection. The operating centre was 45 min away from the laboratory. The slices (400 µm) were prepared from human brain samples (13.61 ± 1.26 mg, n = 16) after surgical interventions using a McIlwain tissue slicer. The slices were stimulated at a supramaximal voltage (2 Hz, 2 ms for 2 min (240 shocks)), as described for rat brain slices. In several cases, the slices were maintained at 8 °C for 20 h under hypoxic conditions to determine whether the catecholamine uptake and release machinery remained intact.

This study was conducted in strict accordance with institutional guidelines, taking the European Community Council Directive (86/609/EEC) into account. The experimental protocol was approved by the Semmelweis University Regional and the Institutional Committee of Science and Research Ethics (No. 116/2015). Informed consent was provided by those patients whose tissue samples were collected during operations and separated for histology.

### Drugs

Levo-[7-^3^H]-noradrenaline (specific activity = 44.5 Ci/mmol, Perkin Elmer, Boston, MA, USA) and 3,4-[7-^3^H]-dopamine (specific activity = 60 Ci/mmol) were purchased from ART (St. Louis MO, USA). KB-R7943 (2-[2-[4(-nitrobenzyloxy)phenyl]ethyl]isothiourea) was obtained from Tocris Bioscience (Ellisville, MO). All other chemicals were obtained from Sigma (Budapest, Hungary).

### Statistical Analysis

The statistical significance of the results was determined by Repeated Measures Analysis of Variance followed by multiple comparison method of Tukey-test. If the measured variables met the normality assumption, two-way factorial measures (FM ANOVA) analysis was performed (see Tables 1 and 3).

**Table 1:** Distribution of [^3^H] activity released from olfactory bulb slices loaded with [^3^H]DA at different temperatures (no MAO inhibitors were used)

[^3^H]	Basal release [%]		Electric stimulation (2 Hz, 240 shocks) [%]	
	37 °C	17 °C	37 °C	17 °C
DA	32.50 ± 2.17 (32.10 ± 2.14 kBq)	26.57 ± 0.49 (22.01 ± 4.06 kBq)	31.67 ± 5.18 (78.01 ± 12.75 kBq)^#^	60.41 ± 2.78*^#^ (138.53 ± 6.37 kBq)^**#**^
DOPAC	37.50 ± 3.13	52.46 ± 3.92^**#**^	24.47 ± 6.20	27.52 ± 2.47
DOPAL	n.d.	n.d.	5.49 ± 0.31*	0.89 ± 0.10*
DOPET	n.d.	n.d.	16.88 ± 3.42*	1.93 ± 0.11*
3-MT	11.91 ± 2.94	12.24 ± 1.72	5.55 ± 2.10	1.67 ± 0.65
HVA	18.10 ± 1.97	8.72 ± 3.83	11.13 ± 3.29	4.28 ± 0.60
Daq	n. d	n.d	4.80 ± 0.82*	3.30 ± 0.11
[^3^H] activity [kBq]	98.77 ± 5.64	82.87 ± 3.90	246.30 ± 42.06^##^	229.32 ± 24.72^##^

*DA* dopamine, *MAO* monoamine oxidase, *DOPAL* 3,4-dihydroxyphenylacetaldehyde, *DOPET* 3,4-dihydroxyphenilethanol, *3-MT* 3-methoxy, 4-hydroxyphenethylamine, *DOPAC* 3,4-dihydroxyphenylacetic acid, *HVA* homovanillic acid, *Daq* dopamine quinone, *n.d.* not detectable

The statistical significance of the results was determined by the TIBC statistical program. To assess the normality of all the continuous variables measured, the Kolmogorov–Smirnov test was used and performed for each individual repeated measurement. If the measured variables met the normality assumption, two-way factorial measures (FM ANOVA) analysis was performed. *significant difference (p < 0.05) between 37 and 17 °C; ^#^p < 0.05 between stimulation evoked and resting release

Note that, at 17 °C, the amount of [^3^H]DA (60.41% of total radioactivity = 138.53 ± 6.37 kBq) is significantly higher than the amount at 37 °C (31.67% = 78.01 ± 12.75 kBq). At 17 °C, the stimulation-evoked release of DOPAL and DOPET was inhibited and the evoked release was enhanced. The release is measured in 3 min collection periods. N = 6

^##^Significant difference (p < 0.05) between the basal and stimulation release values obtained at 17 °C

Student’s t-test was used where appropriate (internal standards). A value of p < 0.05 was considered to be significant. Unless otherwise indicated, the data represent the mean ± S.E. (SEM).

## Results

### Nonvesicular Cytoplasmic Release of DA/NA in Response to Ischemia and Oxidative Stress

Plasmalemmal monoamine transporters exhibit a high degree of sequence homology [66]. Accordingly, noradrenergic/dopaminergic and serotonergic axon terminals are able to take up and release both monoamines (NA and DA) and serotonin [67–69], even in humans [70, 71]. Therefore, the release of DA and NA were measured separately in striatal and cortical slices prepared from rats.

**Fig. 1:**
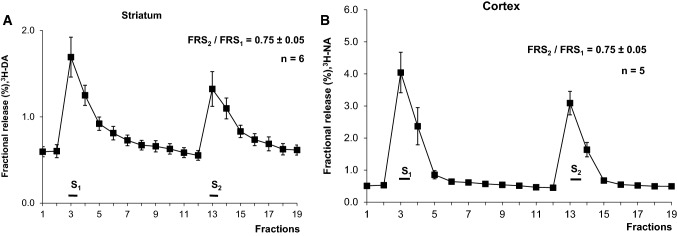
Release of monoamines (DA and NA). **a** The release of [^3^H]dopamine (^3^H-DA) from rat striatal slices in response to stimulation and at rest. The preparations were stimulated during the 3rd (S_1_) and 13th (S_2_) fractions (2 Hz, 2 ms, 240 shocks). The release of [^3^H]DA is expressed as the fractional release (FR). S_1_ = 61,553 ± 9,724 Bq/g (n = 6). The increase by S_1_ p < 0.05 (t-test for Dependent Samples). **b** The release of [^3^H]NA from rat cortical slices following stimulation (2 Hz, 2 ms, 360 shocks). S_1_ = 27,636 ± 1,554 Bq/g (n = 5). The increase by S_1_ p < 0.05 (Wilcoxon Matched Pairs Test). The stimulations are indicated. For further details see “Materials and Methods”

After the rat striatal slices were loaded with [^3^H]DA, the average uptake of radioactivity was 763,000 ± 90,383 Bq/g (n = 6), and the average resting release value during a 3 min collection period was 0.53 ± 0.07% of total radioactivity. Electrical stimulation resulted in the release of radioactivity (S_1_ = 61,553 ± 9724 Bq/g or 1.70 ± 0.25% of total radioactivity), and this stimulated release was repeatable (S_2_): FRS_2_/FRS_1_ = 0.75 ± 0.05 (Fig. 1a). Similar control experiments were performed using [^3^H]NA in cortical slice preparations, where the FRS_2_/FRS_1_ ratio was 0.75 ± 0.05 (Fig. 1b).

**Fig. 2:**
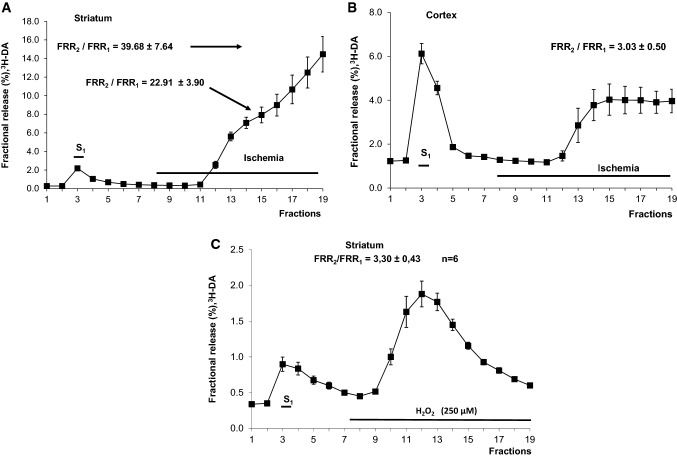
The effects of oxygen and glucose deprivation on the resting release of [^3^H]DA from rat striatal (**a**) and cortical (**b**) slices. The removal of oxygen and glucose (ischemia) was introduced, as indicated, during the 8th fraction and continued until the end of the experiment. The striatal (2 Hz, 2 ms, 240 shocks) and cortical (2 Hz, 2 ms, 360 shocks) slices were stimulated as indicated. n = 6–6. The release of radioactivity is expressed as the fractional release (FR). The effect of ischemia on resting release was expressed as the ratio of the FR values measured at the 7th (R_1_) and at 15th (R_2_)(FRR_2_/FRR_1_ = 22.91 ± 3.90) p < 0.05 (t-test for Dependent Samples) and at the 7th (R_1_) and 19th (R_2_) fractions (FRR_2_/FRR_1_ = 39.68 ± 7.64) (**a**) (t-test for Dependent Samples). In cortical slices FRR_2_/FRR_1_ ratio was calculated measuring FR values at 19th (R_2_) and 7th (R_1_) collection periods (**b**) (t-test for Dependent Samples). 2 For further information, see the “Materials and Methods” section. **c** The effect of oxidative stress (H_2_O_2_, 250 µM) on the resting release of [^3^H]DA from striatal slice preparations (n = 6). H_2_O_2_ was in the Krebs solution as indicated. The administration of oxidative stress started at the 7th fraction and continued until the end of experiments. The release is expressed as fractional release. For experimental conditions, see “Materials and Methods”

**Fig. 3:**
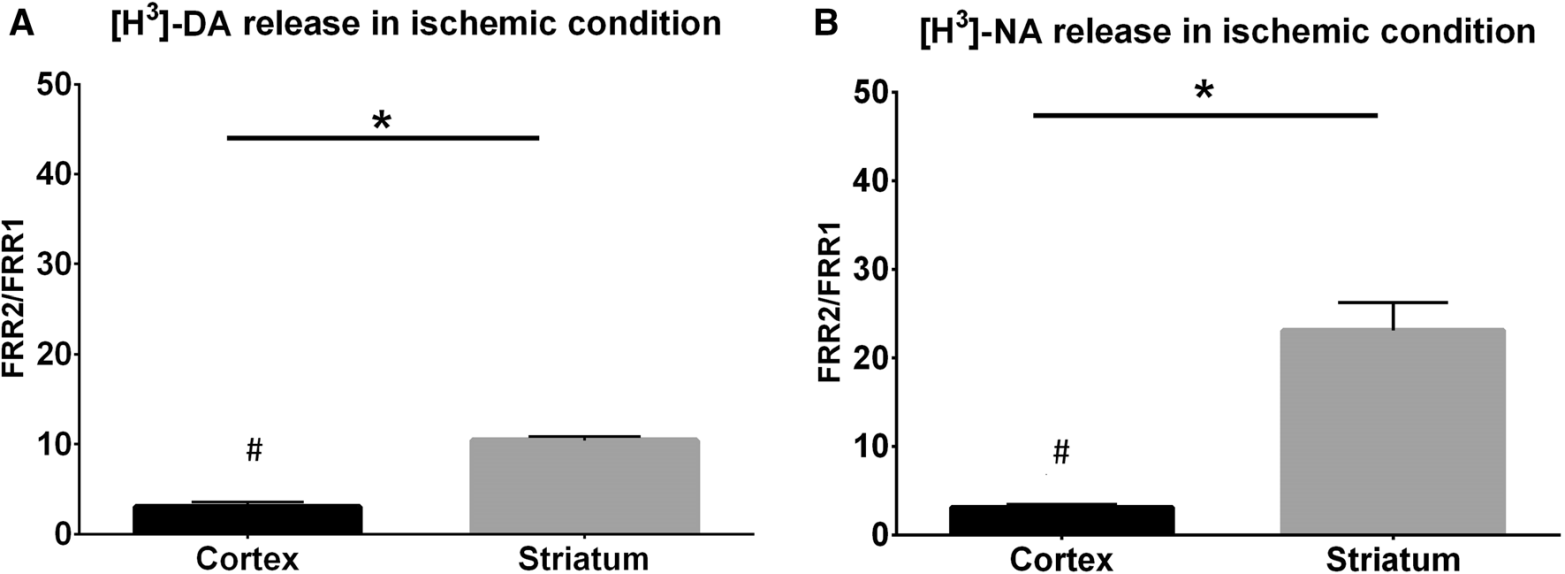
The resting release of [^3^H]DA (A) and [^3^H]NA (B) from rat cortical and striatal slice preparations and the effects of oxygen and glucose deprivation (ischemia) on resting release (n = 6–6). Cortical and striatal slice preparations were exposed to ischemia starting with the 8th fraction and rate of release after 18 min during ischemia at 14th fraction was taken into account (R_2_). Release in 7th fraction is R_1_. For further details, see the “Materials and Methods” section. The effect of ischemia was expressed as FRR_2_/FRR_1_. FRR_1_ was used as an internal control. When either [^3^H]DA or [^3^H]NA release was measured, the effects of oxygen and glucose deficiency were more pronounced in striatal slices than in cortical slices. *p < 0.05, ^#^p < 0.05. Note that these experiments were carried out separately from the experiments shown in Figs. 2, 4 and 5. Repeated measures analysis of variance followed by multiple comparison method of Tukey-test. *p < 0.05

**Fig. 4:**
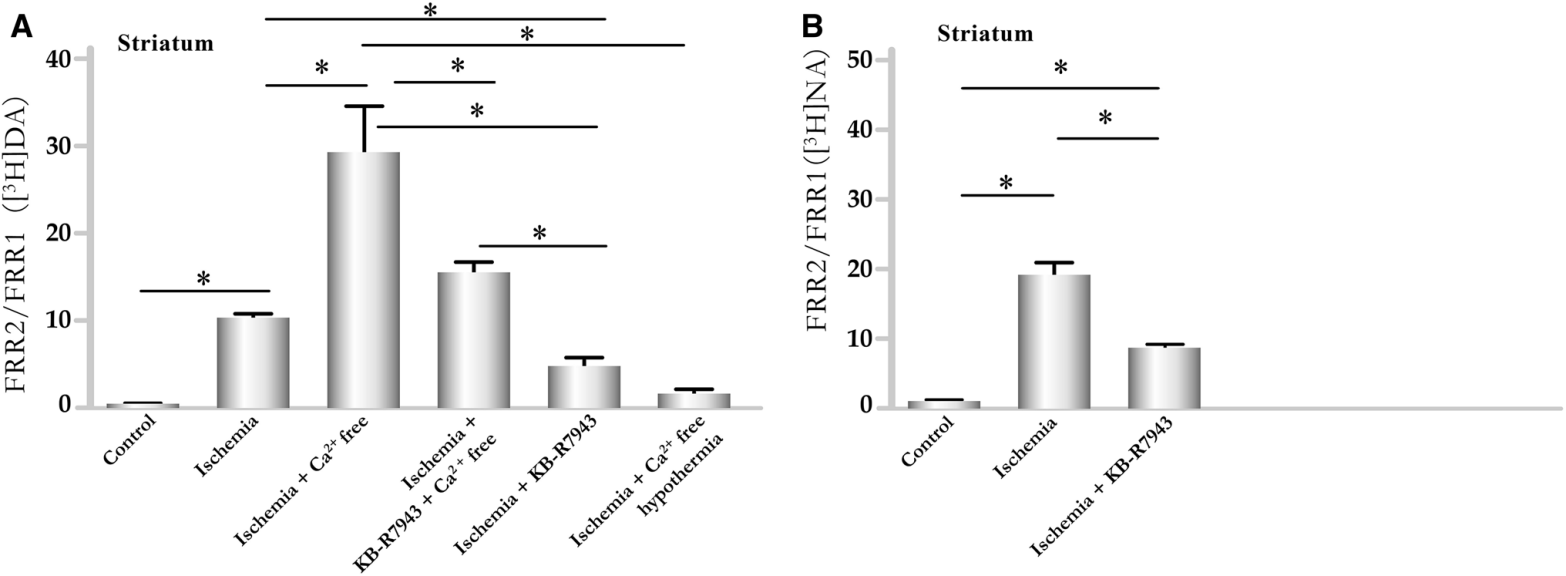
**a** The resting release of [^3^H]DA from rat striatal slices in response to oxygen and glucose deficiency (ischemia) (n = 6) and the potentiation of release by Ca^2+^ removal (n = 6). In the control experiments, FRR_2_/FRR_1_ = 0.91 ± 0.07 (n = 5). The effect of ischemia on resting release was expressed as the ratio of the FR values between the second FR measurement (FRR_2_ noted after 18 min during ischemia) and the first FR measurement (FRR_1_ internal control) (FRR_2_/FRR_1_) of released radioactivity. During hypothermic experiments, the preparations were exposed to a temperature of 17 °C (beginning 30 min before the start of sample collection). KB-R7943 (10 µM) was present in the Krebs solution starting with the 7th collection period and was maintained in the solution throughout the experiments (n = 6). Ca^2+^-free Krebs solution was prepared (Ca^2+^ was removed and 1 mM EGTA was added to the solution starting with the 5th fraction). Note that KB-R7943 significantly reduced the excessive release of [^3^H]DA in response to both ischemia (starting from the 7th collection period) and fully inhibited the release of [^3^H]DA. Repeated measures analysis of Variance followed by multiple comparison method of Tukey-test *p < 0.05. **b **The effect of ischemia on the resting release of [^3^H]NA from striatal slice preparations. KB-R7943 (10 µM) was added to the Krebs solutions starting with the 7th fraction and was maintained in the solution for the remainder of the experiment. The effect of ischemia on resting release was expressed as the ratio between the FR values of the second measurement (FRR_2_, during ischemia) and those of the first measurement (FRR_1_, internal control)(FRR_2_/FRR_1_) of released radioactivity. For condition see legend of **a**. Repeated Measures Analysis of Variance followed by multiple comparison method of Tukey-test). *p < 0.05

**Fig. 5:**
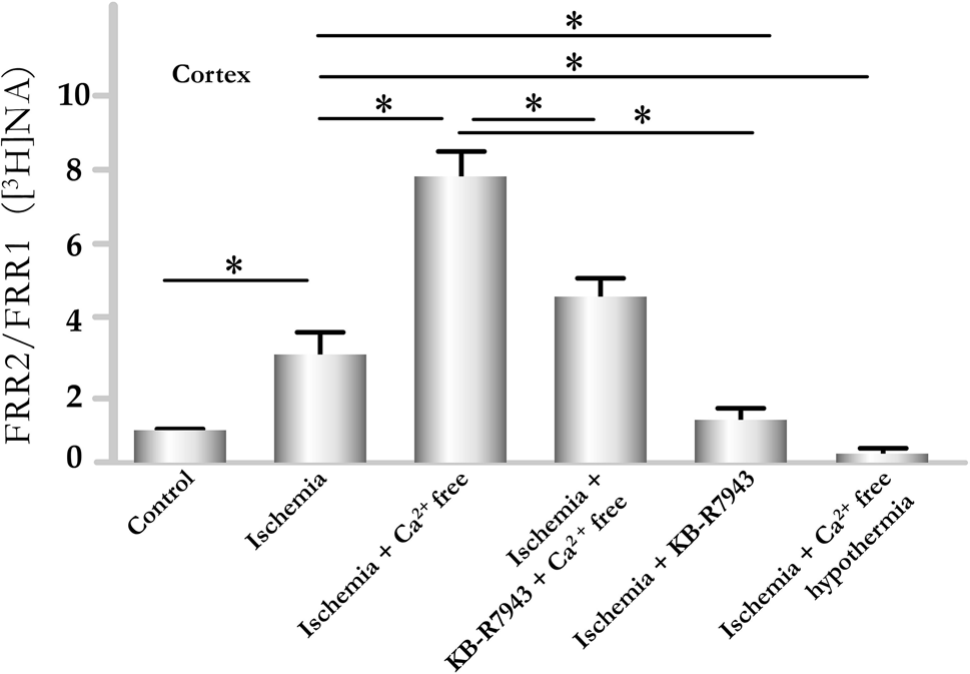
The resting release of [^3^H]NA from rat cortical slices in response to oxygen and glucose deficiency (ischemia) at rest, and the potentiation of release by Ca^2+^ removal. For the control experiment, FRR_2_/FRR_1_ = 0.89 ± 0.60 (n = 5). The preparations were superfused, 3-min fractions were collected, and the effects of ischemia were noted after 18 min (n = 6). KB-R 7943 (10 µM) was present in the Krebs solution starting from the 7th collection and was maintained in the solution throughout the experiments (n = 6). Ca^2+^-free Krebs solution was used (Ca^2+^ was removed and 1 mM EGTA was added to the solution starting with the 5th fraction). Note that KB-R7943 significantly reduced the excessive release of [^3^H]NA in response to both ischemia + Ca^2+^-free solution. Hypothermia (starting from the 7th collection period) also inhibited the [Ca^2+^]_o_-independent resting release of [^3^H]NA. For details, see the legend of Fig. 4. Repeated measures analysis of variance followed by multiple comparison method of Tukey-test. *p < 0.05

The removal of oxygen and glucose gradually increased resting DA release levels from striatal and cortical slice preparations (Fig. 2a, b), resulting in 23-fold (Fig. 2a) and three-fold (Fig. 2b) increases in the resting release level after 21 min, respectively. In the striatal slices the DA release was forty times higher after 33 min Fig. 2a). The effects of ischemic conditions on the resting release levels were considerably weaker in cortical slice preparations than in the striatal slice preparations (compare Fig. 2a, b). In separate experiments the released levels of both monoamines (DA and NA) from striatal slices were measured during ischemic conditions. The release rates were significantly higher than in the released levels of these monoamines from cortical slices (Fig. 3a, b). When supramaximal electrical axonal stimulation was applied during the peak resting release level under ischemic conditions, small insignificant additional increases were observed (data not shown). These findings are consistent with those reported by other studies [72, 73], which showed that the disappearance of synaptic activity is the earliest consequence of cerebral ischemia. In both preparations, when Ca^2+^ ions were removed and 1 mM EGTA was added to the Krebs solution, the resting release levels of both [^3^H]DA (Fig. 4a) and [^3^H]NA (Fig. 5) increased further in response to the combination of oxygen and glucose deprivation.

It is generally accepted that oxidative stress (through H_2_O_2,_ a breakdown product of DA metabolism) caused by ischemia is also involved in brain injury [74]. H_2_O_2_ is a stable molecule both in the intracellular and extracellular space, which makes this ROS nonsynaptically exert its effect of inhibiting stimulation-induced transmitter release [75]. A previous study showed that endogenous glutamate acting on AMPA receptors generates H_2_O_2_ that diffuses and reduces stimulation-evoked release of DA [75]. In our experiments, H_2_O_2_ at a 250 µM concentration increased the non-vesicular resting release of [^3^H]DA (Fig. 2c), producing a threefold increase (FRR_2_/FRR_1_ = 3.30 ± 0.43, n = 6). These findings are consistent with our previous observations [19, 20]. Moreover, in the effluent toxic metabolites (DOPAL, DOPET) and quinone were detected in addition to DA and its metabolites (DOPAC, HVA) (Table 3).

### Effects of NCX Inhibition

The presence of the reverse NCX inhibitor KB-R7943 [41] at a concentration of 10 µM significantly reduced the effects of ischemia on striatal (Fig. 4a, b) and cortical (Fig. 5) slice preparations, as determined by measuring the resting release levels of [^3^H]DA and [^3^H]NA.

When extracellular Ca^2+^ was removed in the presence of KB-R7943, preventing nerve terminals from exchanging Na^+^ for Ca^2+^, the released levels of transmitters induced by ischemia were significantly reduced compared to release under ischemia in Ca^2+^-free Krebs solution (Figs. 4 and 5).

### Effects of Hypothermia

The temperature sensitivity of Na^+^/Cl^−^-dependent monoamine transporters was also demonstrated [76]. The released levels of transmitters evoked by oxygen and glucose removal were significantly reduced in both striatal and cortical slices when the bath temperature was lowered from 37 to 17 °C, even in the absence of extracellular Ca^2+^ (Figs. 4 and 5). This effect is likely due to the temperature dependence of both the uptake process and NCX [77] and provides evidence that the reversed activity of the transporter is responsible for the release of monoamines.

DOPAL is a toxic by-product caused by the oxidation of DA by MAO-B. 3,4-dihydroxyphenilethanol (DOPET) is a subsequent metabolite of DOPAL, produced by aldehyde reductase. No toxic metabolites were detected during resting release, although [^3^H]DA was present in the perfusate obtained from the olfactory bulb slice preparations (Table 1). Electrical stimulation (2 Hz, 240 shocks) enhanced the release of both [^3^H]DA and its toxic metabolites (Table 1). When the temperature was reduced from 37 to 17 °C, the proportion of released [^3^H]DA was increased, and the released levels of [^3^H]DOPAL and [^3^H]DOPET were significantly reduced (Table 1). During DA metabolism, the catechol ring of DA undergoes oxidation and forms a quinone, a toxic metabolite of DA [64] and an ROS [40]. Dopamine quinone (Daq) was also detected in the perfusate when the tissue was stimulated. Interestingly, the release of this metabolite in response to stimulation was not affected by hypothermia (Table 1).

### Effects of Hypothermia on NA Release from Human Cortical Slices

**Fig. 6:**
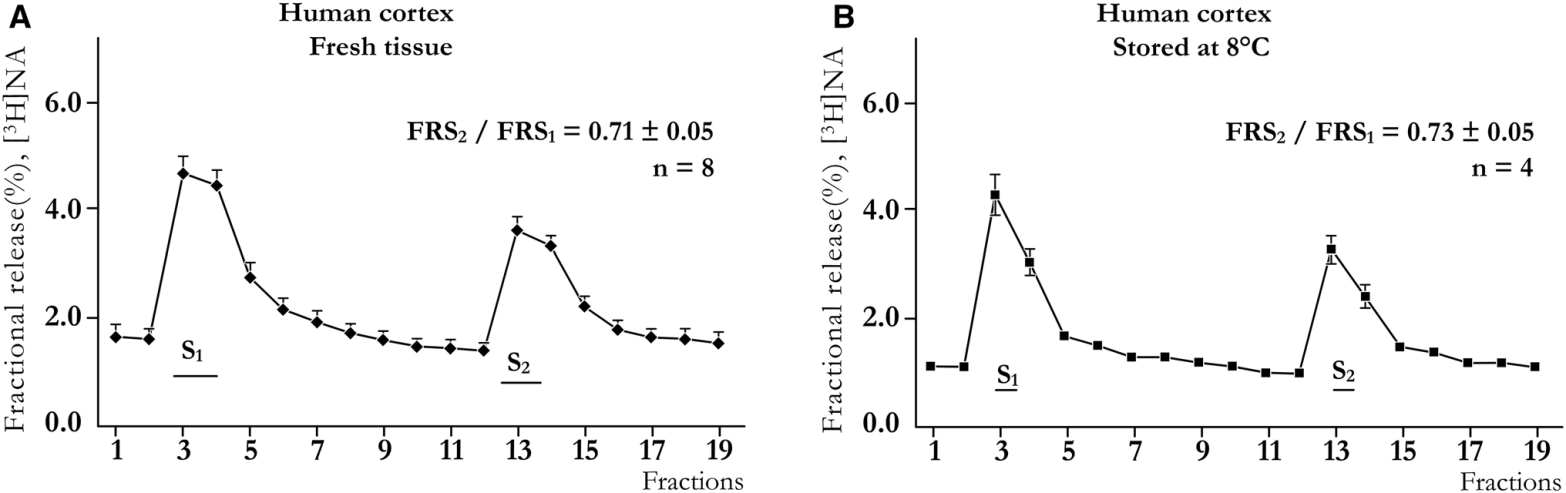
The release of [^3^H]NA from human cortical slices that were either freshly prepared immediately after the operation (**a**) or prepared from tissue maintained at 8 °C for 20 h (**b**) under hypoxic conditions. The release was measured at 37 °C. The slices were stimulated (2 Hz, 2 ms, 240 shocks) twice (S_1_ and S_2_), as indicated. The fractional release (FR) values measured in the samples obtained from the frontal, occipital and parietal cortices were pooled. Increase of [^3^H]NA release evoked by S_1_ p < 0.05 (t-test for Dependent Samples for **a** and **b**)

In our previous study, we showed that the release of transmitters at rest and in response to axonal stimulation can be studied in human cortical slice preparations that are freshly obtained from brain operations [78]. Therefore, to study the preservative effects of hypothermia against brain tissue damage, human frontal cortical slices were maintained at 8 °C for 20 h without oxygen. After rewarming and loading the samples with [^3^H]NA, the uptake and release values did not differ from those measured in tissues that were not exposed to long-lasting hypoxic conditions at low temperatures (Table 2). The release of transmitters that was evoked by stimulation was repeatable in both freshly prepared slices (Fig. 6a) and in slices prepared after being stored at 8 °C under hypoxic conditions (Fig. 6b); this result indicates that even after 20 h, the uptake and release functions were maintained in stored brain tissues. These findings are in accordance with those reported by others that cooling preserves brain tissue and allows it to function for a limited time [79, 80].

**Table 2:** Uptake and stimulation-evoked release of [^3^H]-noradrenaline from human cortical slices (for further information see the “Materials and Methods” section)

	Fresh tissue	Tissue maintained at 8 °C for 20 h	Significance
Uptake (Bq/g)	104,551 ± 8842	119,479 ± 26,467	n.s
	(8)	(6)	
S_1_ (Bq/g)	6991 ± 975	5941 ± 1731	n.s
	(8)	(8)	
S_2_ (Bq/g)	3895 ± 461	3148 ± 372	
	(8)	(2)	
FRS_2_/FRS_1_	0.71 ± 0.04	0.72 ± 0.05	n.s
	(8)	(4)	

Repeated measures analysis of variance followed by multiple comparison method of Tukey-test

## Discussion

Stroke is a leading cause of disability in adults, resulting in social and economic burdens worldwide [81, 82]. Numerous attempts [1, 2, 13, 83–94] have been made to reveal the pathological mechanisms underlying cerebral ischemia and to design new drugs [2, 95–99] that reduce the effects of complications due to stroke [100–102]. Because all of the pharmacological interventions that have been tested to date have failed, new targets for novel therapies are needed.

The human brain is highly metabolically active and accounts for approximately 20% of the oxygen consumption and 25% of the glucose utilization in the body [103]. During chemical neurotransmission, the exocytosis of each vesicle containing transmitters from the presynaptic nerve terminal requires approximately 12,000 ATP molecules [104]. Therefore, the maintenance of functional communications within and among neuronal circuitries in the brain is very sensitive to energy supply deficits. Synaptic transmission failure is the earliest consequence of cerebral ischemia [72, 73].

**Fig. 7:**
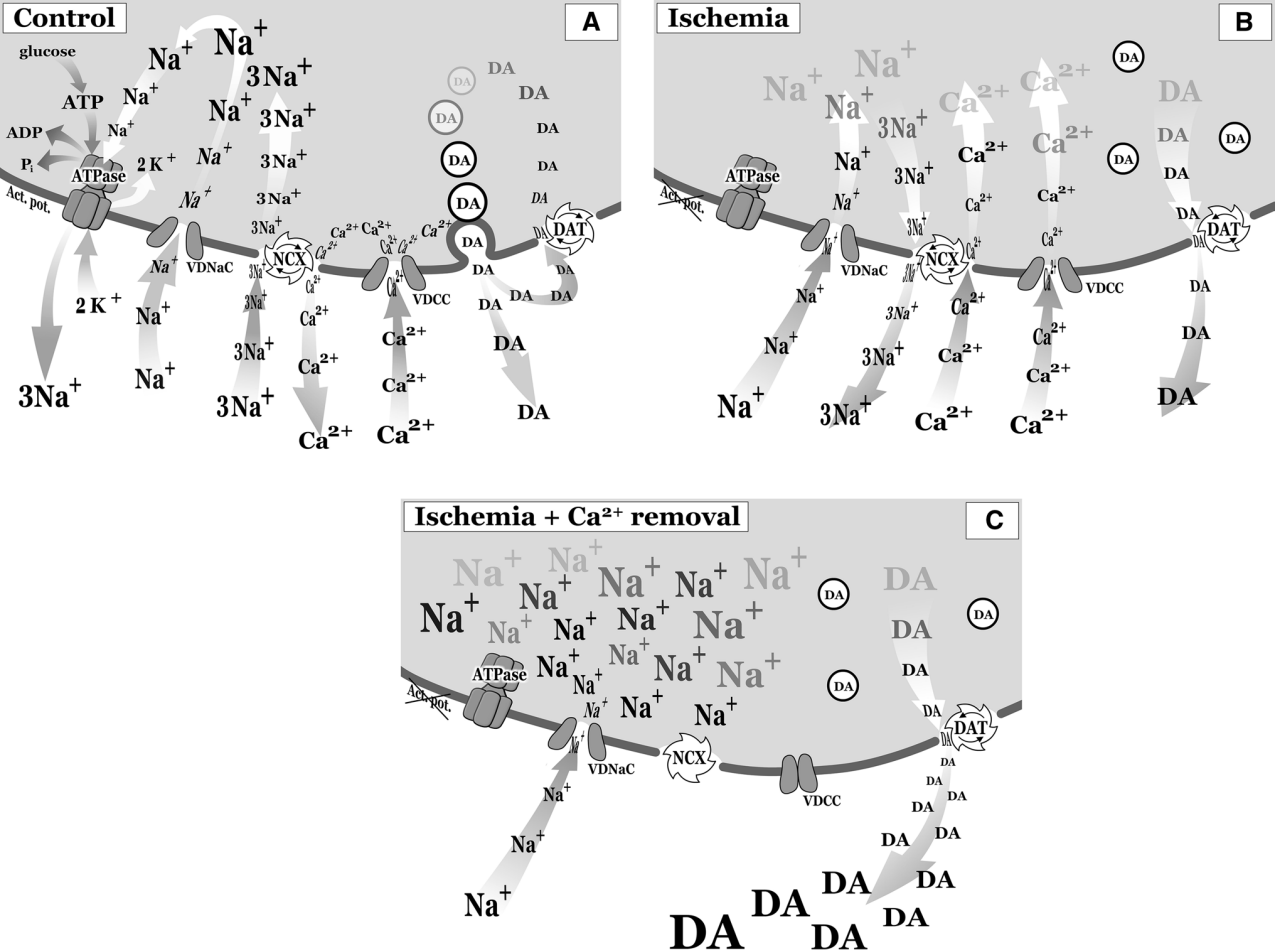
**a** The effects of ischemia on transmitter release (dopamine, DA) and the roles played by the Na^+^/Cl^−^ transporter (DAT) and the Na^+^/Ca^2+^ exchanger (NCX) in the nonvesicular resting release of DA. Dopamine represents an example of various transmitters (NA, serotonine, glutamate, GABA) which under physiological condition stored in vesicles and could be released in response to depolarization followed by Ca^2+^ influx and taken back by Na^+^/Cl^−^ transporters. **a** In response to an action potential (Act. pot.), Na^+^ enters the neuron through voltage dependent sodium channels (VDNaCs), followed by a Ca^2+^ influx through voltage-activated channels (VDCC). The Na^+^/Ca^2+^ exchanger (NCX) is activated when [Ca^2+^]_i_ is ˃ 100 nM. In response to action potentials, [Ca^2+^]_i_ in the nerve terminal can reach concentrations of > 10 µM [32, 33]. The depolarization results in the vesicular release of transmitters (dopamine (DA)). Under physiological conditions, the intraterminal Ca^2+^ levels rise in response to an action potential, and the plasma membrane NCX couples the export of one Ca^2+^ ion to the import of three Na^+^ ions (“forward” mode of NCX activity [28, 77]). Monoamine [108] and glutamate [112] transporters, using ATP as an energy source, operate exclusively in the inward mode, rapidly removing exocytotically released transmitters from the extracellular space and returning them to the nerve terminals to be reused during [Ca^2+^]_o_-dependent vesicular release. **b** Under ischemic conditions, when ATP synthesis is partially or fully blocked [25, 155], the exchanger (NCX) begins to operate in the reverse mode, producing additional Ca^2+^ influx and resulting in Na^+^ efflux. Under this condition, the action potential (Act. pot.) fails, and exocytotic chemical neurotransmission does not occur; the transmitter is released from the cytoplasm by transporters operating in the reverse mode, and the cytoplasmic pool of transmitters (monoamines [23–25], glutamate [23, 26, 156, 157] and GABA [23]) are released. Because ATP is primarily generated by oxidative phosphorylation, the energy supply of the brain depends on a permanent oxygen supply. Any interruption in the oxygen and blood glucose supply, even for only a few minutes, can result in energy delivery failures and cell death [158]. The carrier operates in the reverse mode under the conditions of a reduced Na^+^ electrochemical gradient, such as when nerve terminals become energy compromised during the following events: during ischemia; during the selective inhibition of Na^+^/K^+^-ATPase by ouabain [127, 129, 159]; and after the administration of transporter substrates, such as β-phenylethylamine (β-PEA), mephedrone or amphetamines [46]. The inward transport of substrates by DAT, accompanied by an influx of 2 Na^+^ and 1 Cl^−^ [160], results in a transporter-mediated current [161]. Therefore, in all cases, a high intraterminal [Na^+^] results in the Ca^2+^-independent resting release of catecholamines, their toxic MAO metabolites (aldehydes, DOPAL (3,4-dihydroxyphenylacetaldehyde), quinone and DOPEGAL [133]), in a [Ca^2+^]_o_-independent manner. Glutamate is also released and is involved in neurotoxicity, and a depolarization occurs due to the enhanced [Na^+^]_i_. In addition, a Na^+^ influx through voltage-dependent Na channels (VDNaCs) occurs. **c** Schematic diagram showing that the exchanger (NCX) plays a crucial role in the [Ca^2+^]_o_-independent release of the transmitter DA. When the exchanger is inhibited by a lack of extraneuronal Ca^2+^, it fails to operate in a reverse mode and is not able to reduce [Na^+^]_i_

Rapid synaptic chemical transmission induced in a quantal form requires synchronous vesicle fusion that is evoked by an action potential-induced Ca^2+^-influx (Fig. 7a). However, transmitter release also occurs from the cytoplasm, and this release is independent of action potentials and [Ca^2+^]_o_ [25]. While quantal release is subject to presynaptic modulation, [Ca^2+^]_o_-independent resting release is not affected by presynaptic receptor activation [105]. A prominent feature of cerebral ischemia is the excessive cytoplasmic accumulation of both Ca^2+^ and Na^+^ ions and the excessive production of ROS [21, 22, 106], a condition that can also result in cell death [107].

### Cytoplasmic Release of Catecholamines

In previous studies, it was shown that hypoxic or hypoglycaemic conditions can increase the [Ca^2+^]_o_-independent release of transmitters at rest, including monoamines (DA and NA) [11, 24–26, 108–110] and glutamate [23, 26, 35, 111, 112]. Glutamate released into the extraneuronal space is neurotoxic via the activation of extrasynaptic GluN2B glutamate receptors [113, 114] and the subsequent massive increase in Ca^2+^ influx [115]. Furthermore, H_2_O_2_ produced by glutamate [75, 76] via activation of AMPA receptors is also involved in excitotoxicity. Convincing pharmacological and clinical evidence has demonstrated that the ability of fluoxetine to inhibit GluN2B receptors may have neuroprotective effects [116]. Despite the validation of glutamate toxicity in pharmacological experiments, clinical trials targeting NMDA receptors have failed to achieve beneficial effects [2].

When ischemia was simulated by the removal of oxygen and glucose, the resting cytoplasmic release of catecholamines was enhanced (Figs. 4, 5, and 7b). Ca^2+^ removal potentiated the ischemia-induced resting release of transmitters (Figs. 4 and 5). Similar to our results, the effects of veratridine, a compound that is able to increase [Na^+^]_i_, on GABA release were also potentiated in a [Ca^2+^]_o_-free medium [117]. In both cases, [Na^+^]_I_ is not able to be reduced by NCX operating in reverse, due to the lack of extracellular Ca^2+^ to be exchanged for intracellular Na^+^. When extracellular Ca^2+^ is removed, [Na^+^]_i_ increases further, forcing transporters (NET and DAT) to operate in reverse and resulting in the excessive release of transmitters from the cytoplasm into the extracellular space (Fig. 7c). These neurochemical data are in accordance with observations from neocortical cell cultures [115] showing that neuronal swelling and death were increased by the removal of Ca^2+^ from the ischemic medium.

### Effects of NCX Inhibition

NCX has been shown to be involved in stroke pathophysiology, and its acute and long-term activation reduces brain injury and restores behavioural functions [59]. Furthermore, convincing evidence was obtained demonstrating that KB-R7943 attenuates the elevation of [Ca^2+^]_i_ in response to chemical ischemia [107] and protects CA1 neurons in hippocampal slices against hypoxic and hypoglycaemic injury [41]. These effects also imply that under this condition, [Na^+^]_i_ continues to increase [107]. Based on our study with Ca^2+^ removal, it appears likely that excessive Na^+^ influx, in the absence of NCX-driven Na^+^ efflux results in additional Na^+^ loading and further enhances the [Ca^2+^]_o_-independent cytoplasmic release of transmitters (Fig. 7).

The present study demonstrated that the inhibition of the reverse activation of NCX using KB-R7943 [118–120] preferentially suppressed the resting release of transmitters, which was enhanced by oxygen and glucose removal in both the presence and absence of external Ca^2+^ (Figs. 4 and 5). These findings reveal the role played by the NCX during resting transmitter release. The restricted localizations of the NCX2 and NCX3 isomers primarily to neurons [30] and of NCX1 to cardiac muscle also clarifies the roles played by NCX2 and NCX3 in regulating [Ca^2+^]_i_ and transmitter release in the CNS. Our findings suggest that NCX functioning in reverse mode is involved not only in increasing the level of Ca^2+^ influx but also in increasing the resting release levels of transmitters during ischemia (Fig. 7b). The depolarization caused by high [Na^+^]_i_ further increases Na^+^ influx through the voltage-dependent sodium channel (VDNaC, Fig. 7b, c). Enhanced [Na^+^]_I_ appears likely to trigger transmitter release through a carrier-dependent process. Our findings that hypothermia, a condition that is able to inhibit monoamine transporter function [121], was able to reduce or prevent increased levels of transmitter release (Figs. 4 and 5) strongly supports this mechanism.

Based on our experimental data, the inhibitory effects of KB-R7943 on ischemia-induced transmitter release could easily be ascribed to its inhibitory effects on the reverse operation of NCX. When NCX activity was inhibited by removing Ca^2+^ from the perfusion fluid, KB-R7943 was still able to reduce the ischemia-induced release, a contradiction that requires discussion. Several explanations may explain this discrepancy. KB-R7943 also has several other actions that may be involved in its effects on transmitter release and neuroprotection [28]. The inhibitory effects of KB-R7943 on NMDA channels (IC_50_ = 0.1–11 µM) [122] appears to be one logical explanation for this contradictory effect on transmitter release. Recent evidence suggests that NMDA, but not AMPA receptor antagonists prevented the increased release of transmitters, including those mediated by glutamate and neuronal injury [115]. Furthermore, the fact that Na^+^ channel blockades reduce or inhibit the effects of ischemia on transmitter release [123] supports this explanation. To confirm that the inhibition of the NCX isomer is indeed responsible for the moderation of ischemia-induced transmitter release, selective antagonists, with and without effects on NMDA and Na^+^ channels, are needed that are capable of inhibiting NCX acting in reverse mode. Furthermore, the findings that KB-R7943 inhibits the nicotine receptors expressed in neurons [124] and the noradrenaline transporter [125] also demonstrate the need to study a more selective NCX inhibitor.

The role played by NCX during cerebral ischemia also depends on the anatomical region injured by the insult [28]. In the penumbral region, where the Na^+^/K^+^-activated ATPase is not fully blocked and remains operational, the NCX continues to operate in the forward mode. However, in the ischemic core region, which is primarily represented in our experiments involving oxygen and glucose removal (Fig. 7b), ATP synthesis and the Na^+^/K^+^-activated ATPase are fully blocked; accordingly, [Na^+^]_i_ remains high. When the activity of the Na^+^/K^+^-activated ATPase was inhibited by ouabain, resting transmitter release increased [126–130].

### Protective Effects of Hypothermia on the Release of DA and Its Toxic Metabolites

The beneficial effects of hypothermia on brain function in response to global and local cerebral ischemia have been demonstrated previously [131, 132]. In animal experiments, local short-term cooling after tissue plasminogen activation was shown to protect against side effects, such as oedema, and increased infarct volumes [132]. In our experiments hypothermia inhibited the cytoplasmic release of catecholamines in response to ischemia (Figs. 3, 4, 5) and prevented the release of deaminated toxic DA byproducts (DOPAL) into the extraneuronal space (Tables 1 and 3). These results also provide explanations for the neuroprotective effects of hypothermia.

**Table 3:** Effects of oxidative stress, induced by H_2_O_2_ (250 µM), on the distribution of resting release of [^3^H]DA and its [^3^H] metabolites from striatal slices

[^3^H]	Control resting [%]	Oxidative stress resting H_2_O_2_, 250 µM [%]	Significance
DA	65.64 ± 2.25 63.32 ± 6.11 kBq	50.66 ± 4.69 82.29 ± 7.61 kBq	n.s.
DOPAC	13.99 ± 1.46	10.97 ± 1.12	n.s.
DOPAL	n.d.	3.65 ± 1.07	< 0.05
DOPET	n.d.	8.33 ± 2.73	< 0.05
3-MT	10.49 ± 0.64	10.62 ± 1.30	n.s.
HVA	9.88 ± 0.43	6.07 ± 0.79	< 0.05
Daq	n.d.	9.7 ± 0.97	< 0.05
[^3^H] activity [kBq]	96.48 ± 8.85	162.44 ± 13, 18 kBq	< 0.05

Note that during ischemia, oxidative stress is constantly occurring

The release and distribution of [^3^H]DA and its metabolites were measured using HPLC combined with scintillation spectrometry at the 2nd (control) and 13th (oxidative stress) collection points. Note that the data shown in this Table were not obtained from the experiments shown in Fig. 2c. n = 4

see Table 1, *n.s.* not significant, *n.d.* not detectable

The statistical significance of the results was determined by the TIBC statistical program. To assess the normality of all the continuous variables measured, the Kolmogorov–Smirnov test was used and performed for each individual repeated measurement. If the measured variables met the normality assumption, two-way factorial measures (FM ANOVA) analysis was performed

Under local hypothermic conditions, both the NCX and the uptake transporters become partially or fully inhibited; this inhibition prevents or reduces the excessive carrier-mediated release of transmitters from the cytoplasm and the secondary [Ca^2+^]_i_ elevation caused by the accumulation of [Na^+^]_i_. The level of [^3^H]DA release at 17 °C was significantly enhanced compared to that at 37 °C, although its metabolites were observed at much higher proportions at the higher temperature (Table 1). Gerkau et al. [107] performed in vivo experiments showing that Na^+^ elevation in periinfarct regions results in the reversal of the NCX, triggering a massive secondary [Ca^2+^]_I_ influx while promoting the export of Na^+^. The sustained elevation of [Ca^2+^]_i_ results in the activation of various degradative enzymes, such as phospholipases and proteases. This condition represents a serious risk for neurons. Under this condition, the role of the reversed operation of Na^+^/Cl^−^ transporters is crucial. Hypothermia reduces their operation and prevents transmitters (monoamines and glutamate) from being released into the extracellular space, where they can exert neurotoxic activities, allowing the tissue to recover from an energy-compromised state. Many stroke patients may still fall outside of the clinical time windows for effective treatment. The administration of local hypothermia by emergency services may be an important step, as hypothermia with thrombolysis represents a chance to improve our neuroprotective strategies.

### Toxic Metabolites of Catecholamine Metabolism

Toxic MAO-B metabolites of catecholamines (DOPAL and 3,4-dihydroxyphenylglycolaldehyde, DOPEGAL) can also cause neuronal injury [37, 133]. Dopamine is also metabolized into H_2_O_2_ by MAO-B, and if dopamine is not reduced by cellular antioxidants (GSH and GSH peroxidase), it will react with iron and form hydroxy radicals [134]. In addition, the catechol ring of DA can undergo oxidation and form quinone [64] and H_2_O_2_ + superoxide anions, which are neurotoxic and alter mitochondrial respiration [135]. The energy depletion of mitochondria evoked by ischemic insult results in excessive production of ROS. Mitochondrial oxidative stress is one of the mediators of ischemic brain injury [74].

DOPAL, the toxic metabolite of DA, was detected in the superfusate when [^3^H]DA release was induced by electrical stimulation (Table 1). DOPET, the product of the subsequent metabolism DOPAL by aldehyde reductase, was also enhanced (Table 1). Under these conditions, DA was released from the vesicles. In contrast in our study, we also observed the presence of DOPAL and quinone when DA was released from the cytoplasm under oxidative stress (H_2_O_2_) conditions (Tables 1 and 3). Thus, during ischemia, when dopaminergic axon terminals are energy compromised the resting, non-vesicular release of DA is accelerated. The occurence of neurotoxic DA metabolites (DOPAL and quinone) in the perfusate indicates that they play roles in neuronal toxicity [133, 136, 137].

In our experiments (Fig. 2c), oxidative stress induced by H_2_O_2_, a side product of catecholamine breakdown, resulted in the excessive cytoplasmic release of catecholamines [106] and increased levels of toxic DA metabolites (DOPAL) (Table 3). Furthermore, evidence was obtained that oxidative stress (H_2_O_2_) combined with Na^+^ load drains energy sources, ATP level decreases in the nerve terminal [138, 139]. The importance of our observation with H_2_O_2_ is further supported by findings that the production of ROS is increased in response to stroke and reperfusion [134] and that the presence of H_2_O_2_ at a concentration of 0.1 mM could be detected in the striatum [140]. These findings suggest that ischemia-associated oxidative stress and the toxic metabolites of catecholamines may also be responsible for neurotoxicity.

### Extracellular Space as the Route for Non-synaptic Communication and the Location of Toxic Dopamine Metabolites During Ischemia

Recently, we have provided neurochemical and pharmacological evidence [46] that amphetamine-like drugs of abuse and the trace amine β-phenylethylamine (β-PEA) excessively increase the [Ca^2+^]_o_-independent, non-vesicular release of DA from the cytoplasm into the extracellular space and inhibit the high-affinity transporter. Increases in the DA concentration in the extrasynaptic space tonically control the activity of neurons equipped with DA receptors and are likely to be associated with the reinforcing effects and abusive potential of amphetamines. An overwhelming number of varicosities within the central nervous system are non-synaptically localized [6, 141–143], and they directly release their transmitters into the extracellular space [12, 127, 144]. Therefore, the volume of the extracellular space [145, 146], as an important factor that influences the ambient concentrations of transmitters and their toxic metabolites, and the drugs used in practice that inhabit the extracellular space [6] must also be discussed.

After monoamine transmitters (DA and NA), their toxic metabolites or glutamate have been released into the extracellular space, their diffusion and concentrations are affected by the volume of the space they are released into and their tortuosity [146–150]. During ischemia, a rapid decrease in the volume of the extracellular space and an increase in tortuosity have been reported [151]. These ischemic effects result in the increased concentrations of transmitters released into the extracellular space, augmenting their toxic effects [147]. In addition, Dr. Sykova and her colleagues provided evidence of the roles played by the volume of the extracellular space in various central nervous system diseases [147, 152, 153].

## Summary

The mechanism underlying the ischemia-induced release of transmitters from the cytoplasm involves the inhibition of the Na^+^/K^+^–ATPase enzyme due to energy depletion, the subsequent intracellular Na^+^ accumulation, and the sodium-dependent reversal of the Na^+^/Ca^2+^ exchanger (Fig. 7b) and monoamine uptake carrier [5]. Moreover, the auto-oxidation of catecholamines by the cytoplasmic monoaminoxidase enzyme results in the formation of H_2_O_2_ and toxic metabolites (DOPAL), which are then released into the extracellular space.

Our findings not only suggest the role played by NCX in the reduction of [Na^+^]_i_ under ischemic conditions but also suggest that the brain NCX isoforms, which are able to operate in reverse mode and produce Ca^2+^ overload in neurons, may represent therapeutic targets for the development of new drugs [154]. Moreover, in ischemic insults, local cooling might be a potential treatment for acute stroke intervention, preventing the excessive release of transmitters and their toxic metabolites into the extraneuronal space and lengthening the clinical time windows for effective treatment.
